# Examining Differences Among Opioid Agonist Treatment Clients in Regional and Metropolitan Settings of New South Wales, Australia

**DOI:** 10.1111/ajr.70029

**Published:** 2025-03-19

**Authors:** Daniel T. Winter, Lauren A. Monds, Nicholas Lintzeris, Paul S. Haber, Carolyn A. Day

**Affiliations:** ^1^ Specialty of Addiction Medicine Central Clinical School, Faculty of Medicine and Health, the University of Sydney Sydney New South Wales Australia; ^2^ Edith Collins Centre (Translational Research Centre in Alcohol Drugs and Toxicology), Sydney Local Health District Camperdown New South Wales Australia; ^3^ Drug and Alcohol Services Northern Sydney Local Health District Sydney New South Wales Australia; ^4^ NSW Drug and Alcohol Clinical Research and Improvement Network (DACRIN), Sydney New South Wales Australia; ^5^ Drug and Alcohol Services South Eastern Sydney Local Health District Sydney New South Wales Australia; ^6^ Drug Health Services Sydney Local Health District Camperdown New South Wales Australia

**Keywords:** opioid agonist treatment, opioid use disorder, regional health, substance use, transport, treatment access

## Abstract

**Objective:**

Whilst prior studies have examined characteristics and barriers for opioid agonist treatment (OAT) clients in regional settings, there are limited studies examining these differences in relation to metropolitan OAT clients. This study aimed to examine key characteristics, including sedating medication and substance use, transport and driving behaviours and differences between OAT clients within regional and metropolitan areas of New South Wales, Australia.

**Design:**

Cross‐sectional survey of OAT clients.

**Setting:**

Fifteen public OAT clinics across New South Wales, Australia, between January 2020 and June 2021.

**Participants:**

Survey was completed by 482 people currently receiving OAT.

**Main Outcome Measures:**

Self‐reported sample characteristics, self‐reported sedating medication use and substance use, transportation and driving histories.

**Results:**

Significant differences in OAT pharmacotherapies prescribed between regional and metropolitan participants were noted (aOR = 2.42, 95% CI = 1.42–4.11). Methadone was the most commonly prescribed OAT in both settings (74.1% and 54.4%, respectively). Nearly half (45.6%) of regional participants received OAT from a private dispensary compared to 4.7% in metropolitan areas. While few differences in past‐month substance use were noted, reported heroin use was lower (aOR = 0.27; 95% CI = 0.09–0.78) in regional areas. Regional participants were more likely than metropolitan participants to drive a vehicle to dosing (aOR = 2.89, 95% CI = 1.12–7.46) and less likely to take public transport (aOR = 0.41, 95% CI = 0.18–0.93) or active transport (aOR = 3.75, 95% CI = 1.50–9.40). Few differences regarding driving offences, based on geography, were noted.

**Conclusions:**

Key differences with treatment, substance use, transport and driving were noted within this study. It is evident that regional OAT clients more often rely on motor vehicles to complete daily activities. Such challenges related to OAT should be addressed by informed policy and regulatory changes that ensure access and equity of treatment and safety, regardless of location.

**Summary**

- What this paper adds: what is already known on this subject:
  ○ Opioid agonist treatment (OAT) provides long‐term positive health and social outcomes for people in treatment.
  ○ Client driving safety forms an important clinical consideration for the provision of OAT.
  ○ Barriers to access OAT (and other forms of healthcare) in regional or remote areas frequently include geographic, distance‐based limitations and a lack of transport options.
- What this study adds:
  ○ Differences in the provision of treatment (pharmacotherapy type, dosing point) and substance use between regional and metropolitan clients were evident.
  ○ Regional clients are more reliant on motor vehicles to access treatment and to complete daily activities, with few differences noted in driving offences compared to metropolitan clients.
  ○ Policy and regulatory decisions regarding OAT should consider the unique circumstances of regional and remote clients, ensuring equitable access to treatment.

## 1 Introduction

Within Australia, opioid agonist treatment (OAT) is the standard of care treatment for opioid use disorder and includes methadone, sublingual buprenorphine and depot buprenorphine [1, 2]. OAT has been shown to be an effective medication treatment that improves long‐term health and social outcomes [1, 3], and is a World Health Organisation designated essential medicine [4]. However, access to OAT in Australia varies [2, 5]. For example, while regional and remote areas report higher utilisation of opioid analgesics than metropolitan [6], access to alcohol and other drug treatment services, including OAT, is often limited [5].

Treatment access in regional and remote areas can serve as a limiting factor for treatment uptake and retention of OAT. In part, this may result from a paucity of alcohol and other drug specialists or primary care OAT prescribers, and of community pharmacies or clinics for dispensing, compared to metropolitan areas [7, 8, 9]. For many people receiving OAT, regular attendance at clinical appointments and daily dispensing is required. As such, increased time or distances to travel for treatment, limited access to public transport, and the relatively high cost of owning and maintaining a motor vehicle can further act as barriers to accessing treatment in regional and remote areas [5, 7, 8, 9, 10, 11].

These barriers may be ameliorated by the provision of takeaway doses of OAT, alternate‐day dosing for sublingual buprenorphine, or use of weekly or monthly dosing with depot buprenorphine pharmacotherapies [12, 13]. However, these are first and foremost clinical decisions informed by clients' medical and social history, pharmacological profile and treatment preference.

Additionally, driving safety for those receiving OAT and who have access to a motor vehicle is a clinically important issue. Clinical guidance indicates that clients should not drive a motor vehicle when commencing treatment (i.e., the first 2–4 weeks, depending on the pharmacotherapy prescribed) whilst the dose is titrated to reduce cravings and withdrawals, and a steady state serum drug level has been achieved [13]. Marked changes in dose among established clients can also impact short‐term fitness to drive. The use of potentially impairing substances, including alcohol, illicit drugs, and some prescribed medicines or extramedical use must be considered and managed by clinicians. Consideration of these factors when commencing treatment is essential to ensuring both client and public safety. Further, trends in substance use vary between metropolitan and regional areas. In remote and very remote areas, consumption of opioids and other analgesics is higher compared to metropolitan areas in Australia, and consumption patterns of other drugs (such as cannabis and stimulants) also differ by geographic locations [5, 14].

There is extant literature regarding OAT and clients who receive OAT in regional or rural settings. This includes literature on OAT service delivery [7], client emergency department presentations [15], client treatment characteristics [7] and substance use [8, 16] and key barriers to treatment for rural clients [9]. However, there is limited literature comparing differences between regional and metropolitan OAT clients, particularly in areas such as treatment access, transportation and driving. Albeit some studies have utilised spatial analyses to examine distance and driving times based on census data [17], or geographic disparities of prescribers in regional areas [18].

Indeed, the many barriers that OAT clients face may be compounded by distance and access to transport, which may be mitigated by appropriate provisions to cater for geographic differences. Therefore, this study examined the key differences between regional and metropolitan‐based OAT clients related to (1) client‐specific characteristics, including variations in the provision of OAT, (2) reported substance use, including medications that may cause impairment and (3) client driving and transport behaviours.

## 2 Methods

### 2.1 Study Design and Sample

This study involved a cross‐sectional survey of OAT clients at six regional and nine major city public drug and alcohol clinics across New South Wales (NSW), Australia. Geographic location of clinics and client area of residence was defined by the Australian Statistical Geography Standard for Remoteness [19]. Major city areas as per the Standard are hereafter referred to as metropolitan areas. Eligibility to participate included being aged 18 years or older and a current OAT client at a public drug and alcohol service (i.e., attending the clinic to see their prescriber and/or to receive their OAT medication). Exclusion criteria included insufficient English proficiency and those unable (or unwilling) to consent to study procedures. The survey was administered between January 2020 and June 2021, during periods when COVID‐19 restrictions permitted in‐person researcher attendance.

### 2.2 Procedures

Clients that attended public drug and alcohol clinics were informed of the study prior through printed materials and by clinic staff through word‐of‐mouth discussion. During data collection, staff informed clients of the study and directed them to speak to an on‐site independent researcher if interested in participating. Trained researchers assisted with obtaining verbal informed consent, including discussing that the survey was confidential, anonymous and voluntary. Participants completed surveys by verbal administration with the researcher in a private setting (e.g., a breakout room), taking approximately 10–15 min to complete. All participants received a $20 gift voucher upon completion [20]. The research was approved by the Sydney Local Health District Ethics Review Committee (RPAH Zone; HREC 2019/ETH 10587).

### 2.3 Measures

The survey was developed and administered on the secure web‐based platform, Research Electronic Data Capture (REDCap) [21]. The survey explored participant characteristics (age, gender, employment status, area of residence); OAT details (medication type, dose duration and clinic attendance frequency), and other sedating prescription or other substance use (past month use, referred hereafter as recent use); current transport practices (driving and other transport options, daily time and cost associated with transport) and driving histories (licencing and driving offences, including roadside Mobile Drug Testing, or MDT). The survey tool utilised for this study has been included as File S1.

### 2.4 Statistical Analyses

Data were analysed using IBM SPSS Statistics (version 28.0). Descriptive statistics were summarised for the study variables. Binary logistic regression was used to calculate odds ratios (ORs) and 95% confidence intervals (95% CI) to compare differences based on regional geographic area (i.e., metropolitan, or regional and remote areas), as determined by reported suburb of residence for each participant. Adjusted ORs (aORs) were calculated by the backward elimination (Wald) method to control for age, gender, current OAT medication, place of OAT dosing point and past month heroin use. Significance for all statistical tests was set at *p* < 0.05.

## 3 Results

### 3.1 Demographics

Among the 482 OAT clients who agreed to participate, 21.4% were based in regional areas of NSW. Participants were aged a median of 44.0 years (IQR 37–50 years) and 64.7% identified as male (Table 1).

**TABLE 1:** Summary of demographic characteristics, OAT medication and other substance use histories of the sample, by regional geographic area.

Variable	Total *n* = 482, *n* (%)	Metropolitan^a^, *n* = 379 (78.6%), *n* (%)	Regional and remote^a^, *n* = 103 (21.4%), *n* (%)	Odds ratio (OR)		Adjusted odds ratio (aOR)	
				OR (95% CI)	*p*	aOR (95% CI)	*p*
Age (years), median (IQR)	44 (37–50)	44 (36–51)	44 (37–50)	1.00 (0.97–1.03)	0.872		
Gender^b^							
Female (ref)	167 (34.7)	131 (34.6)	36 (35.0)	0.82 (0.48–1.41)	0.468		
Male	312 (64.7)	245 (64.6)	67 (65.0)				
Non‐binary/other	3 (0.6)	3 (0.8)	—	Exc.			
Primary income source							
Government benefits (ref)	424 (88.0)	340 (89.7)	84 (81.6)	0.94 (0.42–2.11)	0.888		
Paid employment	58 (12.0)	39 (10.3)	19 (18.4)				
Current OAT medication							
Methadone (ref)	337 (69.9)	281 (74.1)	56 (54.4)	**2.40 (1.31–4.11)**	0.004**		
Buprenorphine^c^	145 (30.1)	98 (25.9)	47 (45.6)			**2.42 (1.42–4.11)**	0.001**
Duration on OAT							
Less than 12 months (ref)	109 (22.6)	88 (23.2)	21 (20.4)	0.95 (0.49–1.81)	0.871		
More than 12 months	373 (77.4)	291 (76.8)	82 (79.6)				
Place of OAT dosing							
Public clinic (ref)	417 (86.5)	361 (95.3)	56 (54.4)	**16.87 (8.58–33.19)**	< 0.001***		
Private (pharmacy/clinic)	65 (13.5)	18 (4.7)	47 (45.6)			**16.24 (8.57–30.79)**	< 0.001***
Any substance use, last month^d^							
No (ref)	65 (13.5)	55 (14.5)	10 (9.7)	1.22 (0.42–3.59)	0.713		
Yes	417 (86.5)	324 (85.5)	93 (90.3)				
Sedating Medication use, last month^e^							
No (ref)	180 (37.3)	144 (38.0)	36 (35.0)	1.06 (0.59–2.01)	0.859		
Yes	302 (62.7)	235 (62.0)	67 (65.0)				
Alcohol use, last month							
No (ref)	329 (68.3)	268 (70.7)	61 (59.2)	1.24 (0.70–2.19)	0.456		
Yes	153 (31.7)	111 (29.3)	42 (40.8)				
Heroin use, last month							
No (ref)	390 (80.9)	295 (77.8)	95 (92.2)				
Yes	92 (19.1)	84 (22.2)	8 (7.8)	**0.26 (0.09–0.77)**	0.015*	**0.27 (0.09–0.78)**	0.016*
Stimulant use, last month							
No (ref)	371 (77.0)	282 (74.4)	89 (86.4)				
Yes	111 (23.0)	97 (25.6)	14 (13.6)	0.43 (0.18–1.01)	0.054		
Cannabis use, last month							
No (ref)	239 (49.6)	192 (50.7)	47 (45.6)	1.38 (0.78–2.43)	0.271		
Yes	243 (50.4)	187 (49.3)	56 (54.4)				
Injecting drug use, last month							
No (ref)	346 (71.8)	263 (69.4)	83 (80.6)	2.22 (0.86–5.76)	0.100		
Yes	136 (28.2)	116 (30.6)	20 (19.4)				

^a^ Based on the Australian Standard Geography Standard Remoteness Structure (2016) [19], with metropolitan areas equivalent to major city designation and regional and remote equivalent to all regional and remote designations.

^b^ Data on participants who identified as a non‐binary or other genders (*n* = 3; 0.6%) excluded from the analysis due to sample size.

^c^ Buprenorphine includes sublingual and subcutaneous depot formulations.

^d^ Includes reported use of prescribed or extramedical sedating medications, alcohol and illicit drugs.

^e^ Includes reported use of prescribed or extramedical sedating medications.

Methadone was more likely to be prescribed than buprenorphine in both settings (methadone: 54.4% regional vs. 74.1% metropolitan; buprenorphine: 45.6% regional vs. 25.9% metropolitan), but there were significant differences with a higher proportion of buprenorphine being prescribed in regional settings (aOR = 2.42, 95% CI = 1.42–4.11). Similarly, private dosing sites (including community pharmacies and private clinics) for clients to obtain their OAT were more common in regional areas (45.6%) compared to metropolitan areas (4.7%; aOR = 16.24, 95% CI = 8.57–30.79).

### 3.2 Medication and Substance Use

Reported past month use of any alcohol among participants was noted among the participants (40.8% vs. 29.3%), with no significant differences. While there were no differences regarding illicit drug use broadly, there were notable differences for use of heroin, with lower proportions of regional participants reporting past month use of heroin (7.8% vs. 22.2%; aOR = 0.27, 95% CI = 0.09–0.78). No differences were reported for other illicit drug use based on geography, nor were there any reported differences noted for a range of sedating prescription medications (e.g., benzodiazepines, antidepressants, antipsychotics and pregabalin).

### 3.3 Transportation

Participants reported their primary modes of transport, duration of travel (minutes), and one‐way cost (AUD) to their dosing point in the preceding month (Table 2). Regional clients were significantly more likely to rely on using a motor vehicle (61.4%) compared to those in metropolitan areas (37.7%; aOR = 2.89, 95% CI = 1.12–7.46), and significantly less likely to rely on public transport (11.7% vs. 49.6%; aOR = 0.41, 95% CI = 0.18–0.93). Active transport (i.e., bicycle, walking and other activities that require physical exertion) was also significantly more likely to occur among participants in regional areas (aOR = 3.76, 95% CI = 1.50–9.40) when adjusted for age, gender, current OAT medication, place of OAT dosing and past month heroin use. No differences were noted based on one‐way cost to travel to dosing, nor duration of travel to dosing.

**TABLE 2:** Regular travel mode^a^, estimated one‐way duration, and cost of travel to the dosing point of participants, by regional geographic area.

Mode of transport	Total, *n* = 482	Metropolitan^b^, *n* = 379 (78.6%)	Regional and remote^b^, *n* = 103 (21.4%)	Odds ratio (OR)		Adjusted odds ratio (aOR)^f^	
				OR (95% CI)	*p*	aOR (95% CI)	*p*
Motor vehicle^c^ to dosing, past month^a^							
No (ref)	273 (56.6)	236 (62.3)	37 (35.9)		0.020*		0.029*
Yes	209 (43.3)	143 (37.7)	66 (64.1)	**2.91 (1.18–7.15)**		**2.89 (1.12–7.46)**	
Public transport^d^ to dosing, past month^a^							
No (ref)	282 (58.5)	191 (50.4)	91 (88.3)				
Yes	200 (41.5)	188 (49.6)	12 (11.7)	**0.28 (0.13–061)**	0.001**	**0.41 (0.18–0.93)**	0.032*
Active transport^e^ to dosing, past month^a^							
No (ref)	327 (67.8)	261 (68.9)	66 (64.1)				
Yes	155 (32.2)	118 (31.1)	37 (35.9)	2.04 (0.88–4.76)	0.098	**3.76 (1.50–9.40)**	0.005**
Travel time to dosing, one way (mins), median (IQR)	15.0 (10.0–30.0)	15.0 (10.0–30.0)	10.0 (5.0–20.0)	0.99 (0.97–1.01)	0.365		
Cost to travel to dosing, one way ($AU), median (IQR)	2.50 (0.00–5.00)	2.50 (1.00–5.00)	2.00 (0.00–3.00)	0.95 (0.87–1.04)	0.266		

Abbreviations: $AU, Australian dollars; Mins, minutes.

^a^ More than one response could be provided for each transport mode.

^b^ Based on the Australian Standard Geography Standard Remoteness Structure (2016) [19], with metropolitan areas equivalent to major city designations and regional and remote equivalent to all regional and remote designations.

^c^ Motor vehicle includes a driver and/or passenger of a motor vehicle.

^d^ Public transport includes bus, train/tram, ferry, taxi and ridesharing.

^e^ Active transport includes bicycle riding, walking and other activities that require physical exertion.

^f^ aOR controlled for age, gender, current OAT medication, place of OAT dosing and past month heroin use.

Among those that had recently driven, reasons for driving a motor vehicle included: shopping/errands (82.0%), attending dosing points (79.8%), healthcare appointments (67.2%) and visiting or helping family or friends (68.9%). Regional OAT drivers appeared to be more reliant on driving than their metropolitan counterparts for some of the described daily activities, notably for visiting or assisting family or friends (aOR = 4.33, 95% CI = 1.52–12.34). Whilst driving for transporting children around was significant in univariate analysis (OR = 0.42, 95% CI = 0.18–0.96), this significance diminished when adjusted for age, gender, current OAT medication, place of OAT dosing and past month heroin use (Table 3).

**TABLE 3:** Reason for driving, among participants who reported driving a motor vehicle in the last month, by regional geographic area.

Variable	Total, *N* = 183, *n* (%)	Metropolitan^a^, *n* = 128 (69.9%), *n* (%)	Regional and remote^a^, *n* = 55 (30.1%), *n* (%)	Odds ratio (OR)		Adjusted odds ratio (aOR)^b^	
				OR (95% CI)	*p*	aOR (95% CI)	*p*
To dosing point, last month							
No (ref)	37 (20.2)	30 (23.4)	7 (12.7)		0.709		
Yes	146 (79.8)	98 (76.6)	48 (87.3)	0.81 (2.73–2.42)			
Healthcare appointments, last month							
No (ref)	60 (32.8)	50 (39.1)	10 (18.2)		0.147		
Yes	123 (67.2)	78 (60.9)	45 (81.8)	1.96 (0.79–4.85)			
Work purposes, last month							
No (ref)	145 (79.2)	107 (83.6)	38 (69.1)		0.169		
Yes	38 (20.8)	21 (16.4)	17 (30.9)	1.78 (0.78–4.06)			
Shopping and errands, last month							
No (ref)	33 (18.0)	30 (23.4)	3 (5.5)		0.225		
Yes	150 (82.0)	98 (76.6)	52 (94.5)	2.41 (0.58–9.94)			
To transport children, last month							
No (ref)	132 (72.1)	92 (71.9)	40 (72.7)		0.039*		
Yes	51 (27.9)	36 (28.1)	15 (27.3)	**0.42 (0.18–0.96)**			
Visit or assist family/friends, last month							
No (ref)	57 (31.1)	51 (39.8)	6 (10.9)				
Yes	126 (68.9)	77 (60.2)	49 (89.1)	**4.22 (1.49–11.95)**	0.007**	**4.33 (1.52–12.34)**	0.006**
Other reasons, not described							
No (ref)	111 (60.7)	73 (57.0)	38 (69.1)				
Yes	72 (39.3)	55 (43.0)	17 (30.9)	0.62 (0.30–1.29)	0.200		

^a^ Based on the Australian Standard Geography Standard Remoteness Structure (2016) [19], with metropolitan areas equivalent to major city designation and regional and remote equivalent to all regional and remote designations.

^b^ aOR controlled for age, gender, current OAT medication, place of OAT dosing and past month heroin use.

### 3.4 Driving Offences

Most participants had previously driven a motor vehicle in their lifetime (94.2%), but a much lower proportion reported driving within the previous month (40.3%; Table 4). Among all participants who had ever driven, 70.3% reported lifetime driving offences, with 31.5% reporting a driving offence in the preceding 3 years, with no difference based on regionality. Fifty‐seven participants reported driving under the influence of a substance within the previous month; however, again, with no geographic differences noted. Similar proportions of both regional (35.7%) and metropolitan (38.8%) participants reported mobile drug testing.

**TABLE 4:** Driving history, licensing status and driving offence history of participants who reported ever driving a motor vehicle, by regional geographic area.

Variable	Total *N* = 454, *n* (%)	Metropolitan^a^, *n* = 356 (78.4%), *n* (%)	Regional and remote^a^, *n* = 98 (21.6%) *n* (%)	Odds ratio (OR)		Adjusted odds ratio (aOR)^b^	
				OR (95% CI)	*p*	aOR (95% CI)	*p*
Driven motor vehicle, last month							
No (ref)	271 (59.7)	228 (64.0)	43 (43.9)		0.083		
Yes	183 (40.3)	128 (36.0)	55 (56.1)	1.81 (0.93–3.52)			
Current driver licence							
Learner or no licence (ref)	240 (52.9)	203 (57.0)	37 (37.8)		0.167		
Full or provisional	214 (47.1)	153 (43.0)	61 (62.2)	1.58 (0.83–3.02)			
Licence suspended or cancelled, ever							
No (ref)	171 (37.7)	135 (37.9)	36 (36.7)		0.940		
Yes	283 (62.3)	221 (62.1)	62 (63.3)	1.02 (0.57–1.84)			
Licence suspended or cancelled, last 3 years							
No (ref)	339 (74.7)	261 (73.3)	78 (79.6)		0.073		
Yes	115 (25.3)	95 (26.7)	20 (20.4)	0.53 (0.27–1.06)			
Driven vehicle unlicenced, last 12 months							
No (ref)	373 (82.2)	287 (80.6)	86 (87.8)		0.160		
Yes	81 (17.8)	69 (19.4)	12 (12.2)	0.60 (0.29–1.23)			
Charged with a driving offence, ever							
No (ref)	135 (29.7)	110 (30.9)	25 (25.5)				
Yes	319 (70.3)	246 (69.1)	73 (74.5)	1.16 (0.62–2.20)	0.645		
Charged with a driving offence, last 3 years							
No (ref)	311 (68.5)	245 (68.8)	66 (67.3)				
Yes	143 (31.5)	111 (31.2)	32 (32.7)	1.27 (0.68–2.37)	0.451		
Driving whilst under the influence, last month							
No (ref)	397 (87.4)	318 (89.3)	79 (80.6)				
Yes	57 (12.6)	38 (10.7)	19 (19.4)	1.18 (0.58–2.40)	0.639		
Mobile drug testing (MDT)							
Never tested (ref)	281 (61.9)	218 (61.2)	63 (64.3)				
Tested, negative result only	121 (26.7)	102 (28.7)	19 (19.4)	**0.47 (0.26–0.87)**	0.016*	**0.48 (0.24–0.95)**	0.035*
Tested, ever positive result	52 (11.5)	36 (10.1)	16 (16.3)	1.45 (0.70–3.03)	0.322		

^a^ Based on the Australian Standard Geography Standard Remoteness Structure (2016), [19] with metropolitan areas equivalent to major city designation, and regional and remote equivalent to all regional and remote designations.

^b^ aOR controlled for age, gender, current OAT medication, place of OAT dosing and past month heroin use.

## 4 Discussion

Overall, this study has identified several key differences between regional and metropolitan OAT clients, including medication types, treatment locations and travel to access care, though similarities also emerged. Methadone is most commonly prescribed in both settings, but a higher proportion of regional participants were prescribed buprenorphine pharmacotherapies than in metropolitan areas. Arguably, buprenorphine can afford great flexibility to clients, with the option of alternate day dosing, and a larger allowance of takeaways is available within NSW policy for those receiving sublingual buprenorphine [13]. In the case of depot buprenorphine, once‐weekly or monthly dose options may be particularly advantageous for those seeking greater flexibility or where distance would otherwise preclude treatment [22, 23]. However, treatment options should be driven by a range of clinical decisions and client preferences, and this study did not examine the reasons why buprenorphine is more prevalent in regional areas compared to metropolitan settings.

This study was also undertaken at a time when depot buprenorphine had recently been introduced, and its uptake has since become the majority of buprenorphine treatment in Australia [24]. Additionally, since July 2023, the Australian Government includes OAT under the Pharmaceutical Benefits Scheme (PBS) Section 100 Opiate Dependence Treatment Program, offering a capped cost for treatment. Prior to this, OAT from private clinics and community pharmacies often incurred significant out‐of‐pocket costs, whereas public dosing clinics typically provided OAT at no cost.

We also found some regional differences in reported substance use, with regional participants less likely to report use of heroin. This is comparable to research examining geographic differences at a population level within Australia [5], and with people who inject drugs within NSW [8]. We note that no other substance use differences were reported within this sample when analysed.

Transport habits of OAT clients varied between regional and metropolitan areas, with regional participants more reliant on motor vehicles and active transport and less on public transport. Research on public transport availability in regional and remote areas notes it is often limited compared to metropolitan areas, despite high needs for public transport [25], and can impact access to healthcare or other services [26, 27]. Census data within NSW shows higher public transport use for commuting to work in metropolitan areas (5.0%) compared to regional areas (0.3%–1.3%) [28], whereas Transport for NSW data (which excludes regional areas) indicates a greater use in Greater Sydney (9.5%) compared to regional metropolitan areas (Hunter and Illawarra regions; 3.3% and 4.4%, respectively) [29].

There were no significant differences in duration or cost to dosing between metropolitan and regional areas. Indeed, within both metropolitan and regional areas, clients attending public OAT clinics may not live near the service, which could explain the lack of difference. Additionally, those living further away may not have been recruited or may not currently receive OAT due to geographical barriers, thus not included in the study. Indeed, it is important for health services to understand the diverse geographic and transportation needs of the clients to ensure equitable access to treatment. This study also found no significant differences in most driving offences or licence status among regional and metropolitan participants, with the exception of negative mobile drug testing outcomes.

Recruitment for the survey occurred at public drug and alcohol services across NSW, typically located at public hospitals or health services in major regional towns (i.e., Inner Regional areas) or urban metropolitan areas. Most regional participants were from Inner Regional NSW, with few from Outer Regional or Remote areas and none from Very Remote areas [19]. As such, results from the survey may not be generalisable to OAT clients in more remote areas, where greater barriers to care may exist. Such barriers could be ameliorated through novel models of care, telehealth, increased takeaway doses (where clinically appropriate) and use of depot buprenorphine.

This study represents a large sample of OAT clients across multiple clinics within NSW, including regional locations. Few studies have explored transportation and driving‐related issues for OAT clients, particularly with regional participants. Data were collected via an anonymous, cross‐sectional survey largely based on self‐reported data and verbally administered by a researcher, which may have introduced social desirability or recall bias. To minimise bias, clients completed the survey with independent interviewers not involved in client healthcare and who were trained to provide a non‐judgemental environment [30]. We note other limitations for this study. Recruitment for the study relied on a convenience sample of OAT clients, response rates at each clinic site were unknown, and the survey did not ask participants about dosing schedules. Few clients from very remote areas of NSW were represented in this study; as such, these results may not be representative of OAT clients from these areas. Additionally, as the study was conducted during COVID‐19, where restrictions permitted, the results may no longer reflect current trends, such as for transportation utilisation.

This study provides a greater understanding of key differences between OAT clients in regional or remote areas and metropolitan areas, in particular client and treatment characteristics, reported substance and sedating medication use and transport use and driving histories. These findings have important implications for clinical guidance and clinical practice of OAT in regional settings, particularly around treatment access and availability. Indeed, this study could also help inform broader policy and regulatory changes related to OAT and must consider the impact that they may have on clients and services in regional and remote areas. Understanding the unique needs and circumstances of OAT clients from geographically diverse areas will enable steps to allow equitable and safe access to OAT regardless of geography.

## Author Contributions

**Daniel T. Winter:** conceptualization, data curation, formal analysis, investigation, project administration, writing – original draft. **Lauren A. Monds:** conceptualization, methodology, writing – review and editing. **Nicholas Lintzeris:** conceptualization, methodology, writing – review and editing. **Paul S. Haber:** conceptualization, funding acquisition, methodology, supervision, resources, writing – review and editing. **Carolyn A. Day:** investigation, methodology, supervision, validation, writing – review and editing.

## Ethics Statement

The research was approved by the Sydney Local Health District Ethics Review Committee (RPAH Zone; HREC 2019/ETH 10587).

## Conflicts of Interest

Paul Haber has received research funding from Indivior and Camurus for opioid‐related research and is the recipient of an MRFF/NHMRC Practitioner Research Fellowship. Nicholas Lintzeris has received research funding from Camurus and Indivior for opioid‐related research. All other authors declare no conflicts of interest.

## Supporting information


Appendix S1.

## Data Availability

Research data are not shared.
