# Supplementary material for: Examining Differences Among Opioid Agonist Treatment Clients in Regional and Metropolitan Settings of New South Wales, Australia
Source: Aust J Rural Health. 2025 Mar 19;33(2):e70029. doi: 10.1111/ajr.70029 (PMC11921760; doi:10.1111/ajr.70029)
Supplement: Supplementary file 1 — Appendix S1. [file AJR-33-0-s001.docx]

**SUPPLEMENTARY FILE 1:** Survey instrument for opioid treatment clients (Note, not all variables have been used in within the related manuscript)

SECTION 1 – ABOUT YOU

1. **Age: ___________________**
2. **Gender:**
   - Male
   - Female
   - Non-binary/other
3. **What suburb do you live in? ___________________**
4. **Employment/Income status:**
   - Working/studying part-time
   - Working/studying full-time
   - Disability pension
   - Other government benefits (e.g., Newstart)
5. **What OTP medication are you currently prescribed?**
   - Methadone (Biodone Forte or Methadone Syrup)
   - Buprenorphine (Subutex) or Buprenorphine-naloxone (Suboxone)
   - Buprenorphine injection/depot (Buvidal or Sublocade)
6. **What is your current daily dose of [otp_medication] in mg? ___________________**
7. **If you receive buprenorphine injection/depot, do you receive your dose weekly or monthly?**
   - Weekly
   - Monthly
8. **How long have you been on methadone or buprenorphine treatment?**
   - Less than 1 month (new to treatment)
   - 1 to 3 months (short term)
   - 3 to 12 months (medium term)
   - 1 year and over (long term)
9. **What of the following best describes your usual place of dosing?**
   - Public Clinic
   - Private Clinic
   - Community Pharmacy
10. **What suburb is your place of dosing in? ___________________**
11. **Thinking about the last month, which of the following medication have you taken in addition to your [otp_medication] (regardless of it being prescribed to you or not) (please mark all that apply)**
    - Other Opioids (e.g. Codeine, Fentanyl, Morphine Oxycodone, Tramadol)
    - Benzodiazepines (e.g. Alprax, Normison, Serepax, Valium)
    - Antidepressants (e.g. Celepram, Efexor, Prozac, Zoloft)
    - Antipsychotics (e.g. Abilify, Clozaril, Seroquel, Zeldox, Zyprexa)
    - Pregabalin (e.g. Lyrica, Lypralin, Lyzalon)
    - Gabapentins (e.g. Gabacor, Gapentin, Neurontin, Nupentin)
    - Z-Drugs (e.g. Imrest, Stilnox, Zolpidem, Zopiclone)
    - None of the above
12. **Thinking about the last month, which of the following substances have you taken? (please mark all that apply)**
    - Alcohol
    - Cannabis
    - Amphetamine type substances (e.g. ice, MDMA)
    - Heroin
    - Cocaine
    - None of the above
13. **In previous questions you mentioned you have taken the following medications and substances in the last month. Tick all the ways that you have used these in the last month:**

|  | **Orally/Swallowing** | **Injecting** | **Other** |
| --- | --- | --- | --- |
| **[otp_medication]** |  |  |  |
| Other Opioids (e.g. Codeine, Fentanyl, Morphine Oxycodone, Tramadol) |  |  |  |
| Benzodiazepines (e.g. Alprax, Normison, Serepax, Valium) |  |  |  |
| Antidepressants (e.g. Celepram, Efexor, Prozac, Zoloft) |  |  |  |
| Antipsychotics (e.g. Abilify, Clozaril, Seroquel, Zeldox, Zyprexa) |  |  |  |
| Pregabalin (e.g. Lyrica, Lypralin, Lyzalon) |  |  |  |
| Gabapentins (e.g. Gabacor, Gapentin, Neurontin, Nupentin) |  |  |  |
| Z-Drugs (e.g. Imrest, Stilnox, Zolpidem, Zopiclone) |  |  |  |
| Alcohol |  |  |  |
| Cannabis |  |  |  |
| Amphetamine type substances |  |  |  |
| (e.g. ice, MDMA) |  |  |  |
| Heroin |  |  |  |

1. **In the last month, how do you normally travel to your methadone or buprenorphine doctor appointments? (please mark all that apply)**
   - Bus (as the driver)
   - Car (as a passenger)
   - Motorbike/motor scooter
   - Pushbike/bicycle
   - Train/tram
   - Walk
   - Other ____________________
2. **Please estimate the time it takes you, on average, to get to your methadone or buprenorphine doctor appointments:** ____________________
3. **In the last month, how do you normally travel to your place of dosing? (please mark all that apply)**
   - Bus (as the driver)
   - Car (as a passenger)
   - Motorbike/motor scooter
   - Pushbike/bicycle
   - Train/tram
   - Walk
   - Other ____________________
4. **Please estimate the time it takes you, on average, to get to your place of dosing:** ____________________
5. **On an average day in the last month, estimate how much it would cost you (in Australian dollars) to travel to your dosing point (one-way):** ____________________
6. **Excluding yourself, how many drivers (someone who has a licence and access to a vehicle) are there within your immediate household?**
   - 0
   - 1
   - 2
   - 3
   - 4
   - 5
   - 6
   - 7
   - 8
   - 9
   - 10+

SECTION 2 – DRIVING CHARACTERISTICS

1. **Have you ever driven a car, truck, or a motorbike/scooter?**
   - Yes
   - No
2. **Have you driven in the last month?**
   - Yes
   - No
3. **Have you ever held an unrestricted/full driver licence?**
   - Yes
   - No
4. **What driver licence do you currently hold?**
   - None
   - Learner permit
   - Provisional 1 – Red
   - Provisional 2 – Green
   - Unrestricted/Full Private
   - Unrestricted/Full Commercial
5. **In the last month, what were your reasons for driving? (please mark all that apply)**
   - To transport children to and from school or other activities
   - To drive someone around as a carer
   - To help someone else meet their obligations
   - To get to healthcare appointments (not dosing)
   - For work purposes
   - To go shopping
   - To visit friends or family
   - To drive around for other activities

SECTION 3 – DRIVING HISTORY

1. **In the last 12 months, how often have you driven a vehicle while unlicensed?**
   - Never
   - vehicle while unlicensed? Rarely (< 5 times per year)
   - Often (at least once per month)
   - Regularly (at least once per week)
2. **If you were to drive without a licence, what do you think your chances would be of being caught?**
   - Almost no chance
   - Little chance
   - Not sure
   - Good Chance
   - High chance
3. **Have you ever been charged with any driving offences?**
   - Yes
   - No
4. **If yes to previous question: In the last three years, have you ever been charged with any of the following driving offences?**

|  | **Yes** | **No** |
| --- | --- | --- |
| Driving through a red light |  |  |
| Drink driving (low-, mid-, high-range PCA) |  |  |
| Driving under the influence of drugs |  |  |
| Driving while your licence is suspended, cancelled or refused |  |  |
| Driving while never licenced |  |  |
| Negligent driving occasioning death |  |  |
| Negligent driving occasioning grievous bodily harm |  |  |
| Driving furiously, recklessly, or in a manner dangerous to the public |  |  |
| Speeding LESS THAN 30km/h above the speed limit |  |  |
| Speeding MORE THAN 30km/h above the speed limit |  |  |

1. **Has your licence EVER been suspended or cancelled?**
   - Yes, within the last 3 years
   - Yes, greater than 3 years ago
   - No

SECTION 4 -SUBSTANCE USE HISTORY & DRIVING

1. **In the last month, how often have you driven while under the influence of/feeling the effects of the following substances**

|  | **Never** | **At least once a month** | **At least once a week** | **Daily** |
| --- | --- | --- | --- | --- |
| Alcohol |  |  |  |  |
| Cannabis |  |  |  |  |
| Amphetamine type substances (e.g. ice, MDMA) |  |  |  |  |
| Benzodiazepines (e.g. Alprax, Normison, Serepax, Valium) |  |  |  |  |
| Heroin |  |  |  |  |
| Other Opioids - not prescribed methadone or buprenorphine (e.g. Codeine, Fentanyl, Morphine, Oxycodone, Tramadol) |  |  |  |  |
| Cocaine |  |  |  |  |

1. **On your last occasion of driving while under the influence of/feeling the effect of a substance, which of the following substances had you used? (Select all that apply)**
   - Alcohol
   - Cannabis
   - Amphetamine type substances (e.g. ice, MDMA)
   - Benzodiazepines (e.g. Alprax, Normison, Serepax, Valium)
   - Heroin
   - Other Opioids - not prescribed methadone or buprenorphine (e.g. Codeine, Fentanyl, Morphine, Oxycodone, Tramadol)
   - Cocaine
2. **On your last occasion of driving while under the influence of/feeling the effect of a substance, how much do you think your driving ability was impacted by these substances?**
   - Impaired a lot
   - Impaired a bit
   - No impact or difference
   - Improved a bit
   - Improved a lot
3. **On your last occasion of driving while under the influence of/feeling the effect of a substance, what was the reason you drove? (Select all that apply)**
   - I drove somewhere but did not plan on taking drugs
   - I needed to drive a friend/family member somewhere
   - Medical emergency - I wouldn't normally drive
   - I have problems with mobility and have to drive
   - I had no other way of getting home
   - Too far / more convenient to drive
   - I didn't feel intoxicated
   - Other __________________________
4. **On your last occasion of driving while under the influence of/feeling the effect of a substance, approximately how far (in kilometres) did you drive?**0km -------------------------------------------------------------------------------------------------------- 100km+
5. **On your last occasion of driving while under the influence of/feeling the effect of a substance, estimate the time of the day when you drove:**
   - Morning (6AM to Midday)
   - Afternoon (Midday to 6PM)
   - Evening (6PM to Midnight)
   - Night (Midnight to 6AM)
6. **Have you ever been stopped for Mobile Drug Testing (MDT)?**
   - Yes
   - No
7. **If yes to previous question: Have you receive a positive MDT result for Ecstasy/MDMA, Cannabis, Cocaine, or Methamphetamine?**
   - Yes, in the last month
   - Yes, in the last 12 months
   - Yes, greater than 12 months
   - No
8. **If yes to previous question: For your last MDT, what substances/s were responsible for this positive result? (Select all that apply)**
   - Ecstasy/MDMA
   - Cannabis
   - Cocaine
   - Methamphetamine

SECTION 5 – PERSPECTIVES ON DRIVING RISK

1. **For each of the statements below, please indicate to what extent you agree or disagree:**

|  | **Strongly Agree** | **Agree** | **Undecided** | **Disagree** | **Strongly Disagree** |
| --- | --- | --- | --- | --- | --- |
| People on methadone or buprenorphine should have the same access to a driver licence as ANYONE ELSE. |  |  |  |  |  |
| People on methadone or buprenorphine should have the same access to a driver licence as OTHER PEOPLE ON PRESCRIPTION MEDICATIONS. |  |  |  |  |  |
| People on methadone or buprenorphine should not drive motor vehicles or operate heavy machinery UNTIL THEIR DOSE IS STABLE. |  |  |  |  |  |
| People on methadone or buprenorphine should not drive motor vehicles or operate heavy machinery if USING AN IMPAIRING SUBSTANCE. |  |  |  |  |  |
| I am confident that I can accurately assess MY DRIVING SAFETY & ABILITY |  |  |  |  |  |
| I think that METHADONE impairs my driving ability. |  |  |  |  |  |
| I think that BUPRENORPHINE impairs my driving ability. |  |  |  |  |  |

SECTION 6 – ACCESS TO ADVICE & INFORMATION

1. **Consider each of the statements below and if they apply to you:**

|  | **Yes** | **No** | **Don’t recall/ Unsure** |
| --- | --- | --- | --- |
| I have been informed by my OTP DOCTOR about safe driving for people on methadone or buprenorphine. |  |  |  |
| I have been informed by DOSING STAFF about safe driving for people on methadone or buprenorphine. |  |  |  |
| have been informed by OTHER STAFF (e.g. nurse/case  worker/counsellor) about safe driving for people on methadone or buprenorphine. |  |  |  |
| I have seen WRITTEN INFORMATION about safe driving for people on methadone or buprenorphine. |  |  |  |

SECTION 7 – FINAL THOUGHTS

1. **From your experience what are the greatest risks or concerns around driving safety for people on methadone or buprenorphine?** ______________________________________
2. **What can be done to improve your driving safety and the safety of other people who are on methadone or buprenorphine?** ______________________________________
3. **Did you have any final comments or perspectives that you would like to mention relating to driving safety for people on methadone or buprenorphine?** ______________________________________
